# Molecular and genetic basis of plant architecture in soybean

**DOI:** 10.3389/fpls.2024.1477616

**Published:** 2024-10-07

**Authors:** Weiwei Li, Lei Wang, Hong Xue, Mingming Zhang, Huan Song, Meng Qin, Quanzhong Dong

**Affiliations:** Keshan Branch of Heilongjiang Academy of Agricultural Sciences, Qiqihar, China

**Keywords:** soybean, plant architecture, stem growth habit, internode length, branch, leaf architecture

## Abstract

Plant architecture determines canopy coverage, photosynthetic efficiency, and ultimately productivity in soybean (*Glycine max*). Optimizing plant architecture is a major goal of breeders to develop high yield soybean varieties. Over the past few decades, the yield per unit area of soybean has not changed significantly; however, rice and wheat breeders have succeeded in achieving high yields by generating semi‐dwarf varieties. Semi-dwarf crops have the potential to ensure yield stability in high-density planting environments because they can significantly improve responses to fertilizer input, lodging resistance, and enhance resistance to various abiotic and biotic stresses. Soybean has a unique plant architecture, with leaves, inflorescences, and pods growing at each node; internode number greatly affects the final yield. Therefore, producing high-yielding soybean plants with an ideal architecture requires the coordination of effective node formation, effective internode formation, and branching. Dozens of quantitative trait loci (QTLs) controlling plant architecture have been identified in soybean, but only a few genes that control this trait have been cloned and characterized. Here, we review recent progress in understanding the genetic basis of soybean plant architecture. We provide our views and perspectives on how to breed new high-yielding soybean varieties.

## Introduction

Soybean (*Glycine max* [L.] Merr.) is an economically important crop, and provides approximately one-quarter of the world’s plant protein for food and animal feed (Graham and Vance, 2003; Carter et al., 2004; Wilson, 2008; Hartman et al., 2011). Cultivated soybean was domesticated from wild soybean (*G. soja* Sieb. & Zucc.) approximately 5000 years ago in China, and subsequently spread worldwide (Carter et al., 2004; Wilson, 2008). Soybean yield is ultimately determined by the number of seeds per unit area and seed mass, both of which are affected by number of internodes, branches, pods per plant, seeds per pod, seed size, and plant height (Pedersen and Lauer, 2004; Liu et al., 2020). In addition, soybean yield also is affected by the angle of petiole and length of petiole, both of which are associated with canopy structure and photosynthetic efficiency (Gao et al., 2017; Liu et al., 2020; Zhang et al., 2022). Soybean yield component traits are significantly correlated with both phenotype and genotype (Zhang et al., 2015).

Plant architecture is an essential target trait for developing high-yielding soybean cultivars. This trait can be altered by modulating genes that control stem growth habit, node number, internode length, branch number, leaf size and shape, and leaf angle (Hartung et al., 1981; Bao et al., 2019; Sun et al., 2019; Chen et al., 2021). In the past decade, many quantitative trait loci (QTLs) controlling important agronomic traits have been identified in soybean, some of which have been integrated into the soybase database (https://www.soybase.org/). However, only a small number of the responsible genes for these QTLs have been cloned and functionally characterized. Here, we focus on the genes that have been functionally validated (**Table 1**).

**Table 1:** Genes of published in soybean plant soybean plant architecture.

Trait	Name	Gene ID	Conserved domain or function	Alleles	References
Stem growth habit	*Dt1*	*Glyma.19G194300*	Terminal flower 1b	*Dt1*, *dt^ab^*, *dt^bb^*, *dt^ta^*, *dt^tb^*	Liu et al., 2010; Tian et al., 2010
	*Dt2*	*Glyma.18G273600*	MADS-domain transcription factor	*Dt2*, *dt2*	Ping et al., 2014
Internode length	*DW1*	*Glyma.08G163900*	Key enzyme entkaurene synthase	*DW1*, *dw1*	Li et al., 2018
	*CRY1/2*	*CRY1a* (*Glyma.04G101500*) *CRY1b* (*Glyma.06G103200*) *CRY1c* (*Glyma.14G174200*) *CRY1d* (*Glyma.13G089200*) *CRY2a* (*Glyma.10G180600*) *CRY2b* (*Glyma.02G005700*) CRY2c (*Glyma.20G209900*)	Cryptochromes		Lyu et al., 2021
	*STF1/2*	*STF1* (*Glyma.18G117100*) *STF2* (*Glyma.08G302500*)	bZIP transcription factor		Lyu et al., 2021
	*GA2ox7a/7b*	*GA2ox7a* (*Glyma.20G141200*) *GA2ox7b* (*Glyma.11G003200*)	Gibberellin 2-oxidase		Lyu et al., 2021
	*GA2ox8A/8B*	*GA2ox8A* (*Glyma.13G287600*) *GA2ox8B* (*Glyma.13G288000*)	Gibberellin 2-oxidase		Wang et al., 2021
	*LHY1/2*	*LHY1a* (*Glyma.16G017400*) *LHY1b* (*Glyma.07G048500*) *LHY2a* (*Glyma.03G261800*) *LHY2b* (*Glyma.19G260900*)	MYB domain transcription factor	*Tof16*, *tof16-1*(*lhy1a-1*), *tof16-2* (*lhy1a-2*)	Cheng et al., 2019; Dong et al., 2021
	*RIN1*	*Glyma.12G224600*	SPA family protein	*rin1*	Li et al., 2023
	*PH13*	*Glyma.13G276700*	SPA family protein	*PH13^H3^*	Qin et al., 2023
Branch number	*miR156b*	*Glyma.14G013200*	*MicroRNA156b*		Sun et al., 2019
	*SPL9*	*SPL9a* (*Glyma.02G177500*) *SPL9b* (*Glyma.09G113800*) *SPL9c* (*Glyma.03G143100*) *SPL9d* (*Glyma.19G146000*)	Squamosa promoter binding protein-like (SPL) transcription factors		Bao et al., 2019
	*Dt2*	*Glyma.18G273600*	MADS-domain transcription factor	*Dt2, dt2*	Liang et al., 2022
	*SOC1a*	*Glyma.18G224500*	MADS-domain transcription factor	*Tof18^A^* (*SOC1a^A^*), *Tof18^G^* (*SOC1a^G^*)	Liang et al., 2022; Kou et al., 2022
Leaf architecture	*Ln*	*Glyma.20G116200*	JAGGED transcription factor	*Ln*, *ln*	Jeong et al., 2012
	*ILPA1*	*Glyma.11G026400*	APC8-like protein	*ILPA1*, *ilpa1*	Gao et al., 2017
	*PIN1*	*PIN1a* (*Glyma.08G054700*) *PIN1b* (*Glyma.07G102500*) *PIN1c* (*Glyma.09G30700*) *PIN1d* (*Glyma.03G126000*) *PIN1e* (*Glyma.19G128800*)	Pinformed 1		Zhang et al., 2022

## Genetic basis of stem growth habit

Stem growth habit is a major agronomic trait affecting soybean seed yield because it is related to plant height, flowering time, maturity, abiotic stress tolerance, root architecture, node production (Bernard, 1972; Specht et al., 2001; Heatherly and Smith, 2004; Zhang et al., 2019). Semi-dwarf soybean plant is one of the most important target traits for enhancing lodging resistance and improving yield. Over the past few decades, great efforts have been done to improve soybean yields by stem growth habit-based selection for a semi-dwarf soybean plant (Liu et al., 2010; Tian et al., 2010; Ping et al., 2014). It has been demonstrated that stem growth habit is controlled by two classical genetic loci *Dt1* and *Dt2* in soybean (Bernard, 1972; Ping et al., 2014). *Dt1Dt2* genotypes produce semi-determinate phenotypes, *Dt1dt2* genotypes produce indeterminate phenotypes, *dt1Dt2* and *dt1dt2* genotypes produce determinate, indicating that the *dt1* allele has an epistatic effect on the *Dt2/dt2* locus.

*Dt1* encodes a *TERMINAL FLOWER 1* (*TFL1*) protein in soybean (Liu et al., 2010; Tian et al., 2010). It has been showed that the transition from indeterminate to determinate stem growth habit was caused by independent human selection of four distinct single-nucleotide substitutions in the coding sequence of *Dt1* gene during soybean domestication, each of which led to a single amino acid change that resulted in a recessive *dt1* allele specifying determinate stem growth (Tian et al., 2010). *Dt2* encodes a gain-of-function MADS-domain transcription factor belonging to the APETALA (AP1)/SQUAMOSA subfamily in soybean (Bowman et al., 1993; Gu et al., 1998; Ferrandiz et al., 2000; Ping et al., 2014). *Dt2* interacts with *SUPPRESSOR OF OVEREXPRESSION OF CONSTANS 1* (*SOC1*) in the shoot apical meristem, where they directly bind to the promoter of *Dt1* to repress its transcription and modulate the semi-determinate growth habit in soybean (Liu et al., 2016). Recently, a third locus *Dt3* that controlling soybean stem growth habit was discovered, and confirmed that recessive allele *dt3* was responsible for semi-determinate stem growth habit in soybean (Clark et al., 2023).

## Genes responsible for internode length

Plant height is a key plant architecture trait that directly affects lodging resistance and soybean yield (Chapman et al., 2003; Liu et al., 2020). Internode length and main stem node number determine plant height in soybean (Liu et al., 2013; Chang et al., 2018). Reduced plant height due to shortened stems is beneficial for improving crop yield potential, increasing resilience to abiotic stress, and the use of agronomic and management practices for rapid crop production (Peng et al., 1999; Hedden, 2003; Liu et al., 2020; Lee et al., 2022). A shorter stem due to shortened internodes is typically observed in plants deficient in endogenous gibberellin (GA) biosynthesis or defective in the perception of GA (Yamaguchi, 2008; Binenbaum et al., 2018).

In soybean, *DWARF MUTANT 1* (*DW1*) encodes an ent-kaurene synthase, a key enzyme in the GA biosynthetic pathway that plays a crucial role in GA-regulated cell elongation in stem internodes (Li et al., 2018). The *dw1* mutant shows reduced bioactive GA contents, resulting in a dwarf phenotype (Li et al., 2018). Overexpressing the cryptochrome genes *CRY1s* increased the abundance of STF1 and STF2 proteins, which directly activated the expression of *GA2ox* genes to deactivate GA_1_ and repress stem elongation (Lyu et al., 2021). Meanwhile, overexpressing *gibberellin 2-oxidase 8* genes (*GA2ox8A* and *GA2ox8B*) reduced bioactive GA contents to decrease internode and suppress trailing growth (Wang et al., 2021). Meanwhile, there is a strong artificial selection in cultivated soybean in the genomic region of *GA2ox8A* and *GA2ox8B* (Wang et al., 2021).

A quadruple mutant of soybean *LATE ELONGATED HYPOCOTYL* (*LHY*) genes exhibited reduced expression of GA pathway genes, reduced plant height, and shortened internodes (Cheng et al., 2019; Dong et al., 2021). In addition, multiple genes involved in regulating plant height by shortening internode length have been reported. For example, overexpression of *GmMYB14* transgenic soybean plants shows reduced plant height, internode length, leaf area, and leaf petiole length and angle as well as improved soybean yield when grown in the field under high-density conditions (Chen et al., 2021). Recent research shows that two homologous *SUPPRESSOR OF PHYA* (*SPA*) genes *Plant Height 13* (*PH13*) and *reduced internode 1* (*rin1*) play an important role in regulating internode length in soybean. Loss-of-function of *RIN1* and *PH13* significantly reduced internode length and enhanced grain yield under high-density planting conditions in field trials (Li et al., 2023; Qin et al., 2023).

## Molecular basis of branch number

Shoot architecture plays a pivotal role in determining high-yielding crops, and shoot branching is a major component of shoot architecture (Mathan et al., 2016; Barbier et al., 2017). Meanwhile, shoot branching also plays an important role in controlling soybean yield (Liang et al., 2022), and modulating branch number is crucial for high-yield soybean breeding (Liu et al., 2020). Shoot branching is an agronomically important and complex developmental trait controlled by a group of genes and influenced by environment and genotype × environment interactions. Genome-wide analysis using homology searches identified 406 genes that might be associated with branching in soybean, 57 of which colocalize with QTLs for soybean branching (Tan et al., 2013). However, to date, few genes associated with soybean branching have been described.

Overexpressing *miR156b* in soybean significantly increased the number of long branches and the 100-seed weight, resulting in a 46%–63% increase in yield per plant (Sun et al., 2019). *GmmiR156b* regulated plant architecture by directly cleaving the *SQUAMOSA PROMOTER BINDING PROTEIN-LIKE9d* (*SPL9d*) transcript. SPL9d physically interacted with the homeobox protein WUSCHELa/b (WUSa/b) to regulate axillary bud formation and shoot branching (Sun et al., 2019). The soybean genome contains four *SPL9* homologs (*SPL9a*, *SPL9b*, *SPL9c*, and *SPL9d*), all of which are negatively regulated by *GmmiR156b* (Cao et al., 2015; Sun et al., 2019). The s*pl9abcd* homozygous quadruple mutant of Williams 82 has more branches and nodes than the wild type (Bao et al., 2019). Dt2 interacted with Agl22 and SOC1a to bind the promoters of *Ap1a* and *Ap1d* to activate their transcription, resulting in reduced branching (Liang et al., 2022). In addition, Overexpression of *GmMYB181* could increase the branch number in *Arabidopsis* (Yang et al., 2018).

## Critical genes for leaf architecture

Leaf architecture affects photosynthetic efficiency, canopy coverage, and ultimately plant productivity in many legume crops (Gao et al., 2017). Leaf growth direction is controlled by the curvature of the petiole, which is defined as the angle between the leaf petiole and the main stem (Rodrigues and MaChado, 2008; Gao et al., 2017). A few genes that control leaf shape and leaf petiole angle in soybean have been identified.

Leaves and flowers develop continuously at the flanks of the shoot apical meristem in flowering plants. A single mutation often causes pleiotropic phenotypes during leaf and flower development (Tsukaya, 2006), suggesting that a common regulatory circuit is involved in the production of leaves and flowers. One major *Ln* locus that contributes to the variation in leaflet and seed number per pod (Domingo, 1945; Tischner et al., 2003). Broad leaflets are usually associated with non-4-seeded pods, and narrow leaflets are linked to 4-seeded pods. Broad leaflets and non-4-seeded pods are thought to be dominant over narrow leaflets and 4-seeded pods (Domingo, 1945; Jeong et al., 2011). It has been demonstrated that *Ln* encodes JAGGED1 (JAG1) protein, which regulates lateral organ development; variants of *JAG1* have pleiotropic effects on fruit patterning (Dinneny et al., 2004; Ohno et al., 2004; Jeong et al., 2012; Fang et al., 2013). The transition from broad (*Ln*) to narrow leaflets (*ln*) is associated with an amino acid substitution in the EAR motif of JAG1 (Jeong et al., 2012; Fang et al., 2013).

Leaf petiole angle is particularly important for determining plant architecture in soybean and many other legumes (Rodrigues and MaChado, 2008; Zhou et al., 2012). A soybean mutant *Increased Leaf Petiole Angle1* (*ilpa1*) with increased leaf petiole angle is a gamma ray-induced mutant derived from Chinese soybean cultivar Hedou 12 (Song et al., 2015). The *ILPA1* locus encodes an APC8-like protein that functions as a subunit of the anaphase-promoting complex/cyclosome. Loss-of- function alleles *ILPA1* lead to leaf development defects and alter petiole angle by promoting cell proliferation (Gao et al., 2017).

The auxin signaling regulators Auxin/Indole‐3‐Acetic Acid (Aux/IAA) and Auxin Response Factor (ARF), the auxin co‐receptor Transport Inhibitor Response1/Auxin‐related F‐box Protein (TIR1/AFB), and the auxin‐conjugating enzyme Gretchen Hagen 3 (GH3) all influence the establishment of petiole angle in monocots (Song et al., 2009; Bian et al., 2012; Zhao et al., 2013; Chen et al., 2018; Liu et al., 2018). The auxin efflux transporter genes *PINFORMED1a* (*PIN1a*) and *PIN1c* determined polar auxin transport and controlled plant architecture and petiole angle in soybean. The *pin1abc* triple mutant shows a semidwarf stature and a small leaf petiole angle (Zhang et al., 2022). Meanwhile, (Iso)flavonoids inhibit the transcript of *PIN1a/c* to regulate petiole angle in soybean (Zhang et al., 2022).

## Conclusions and perspectives

**Figure 1:**
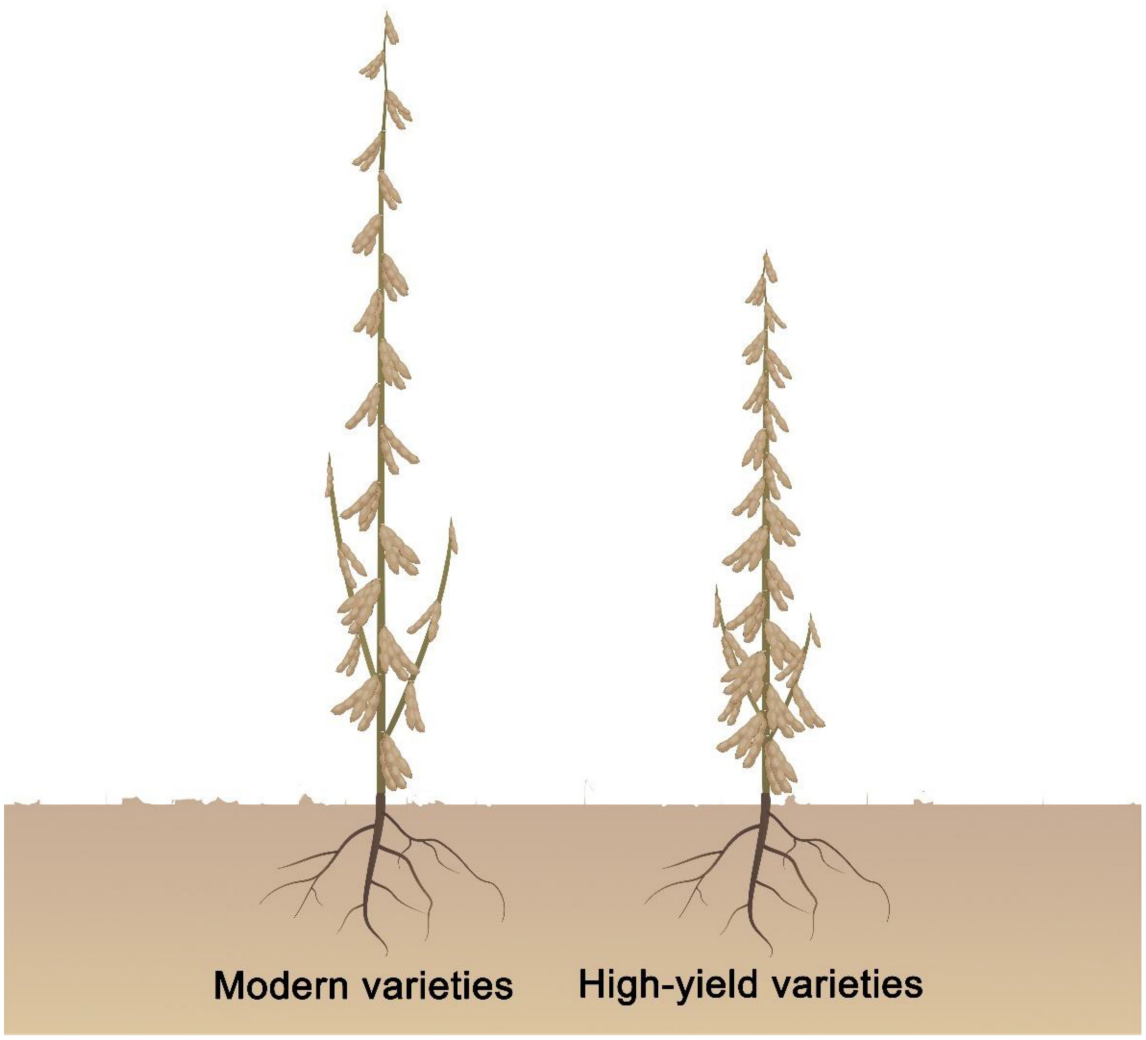
Proposed high yield and ideal plant architecture in soybean. High yield soybean varieties should have shorter internode length, more internodes, lodging tolerance, narrow leaflet and higher the ratio of four seed per pod, smaller petiole angle, and shorter petiole, few or no short branches, tolerate high‐density planting.

Plant architecture plant critical role in affecting crop production (Huyghe, 1998; Jiao et al., 2010; Miura et al., 2010; Tian et al., 2019). The application of semi dwarf varieties has significantly improved crop yield by increasing the planting density and the lodging resistance. The gains in grain productivity during the Green Revolution were a direct consequence of optimal plant height. Mutant alleles of the Green Revolution genes *Semidwarf1* (*Sd1*) and *Reduced height* (*Rht*) are utilized to improve crop yields by decreasing overall plant (Peng et al., 1999; Sasaki et al., 2002). In addition, maize cultivars with more upright leaf angles can produce more grains per unit land area when grown in the field under high-density conditions (Lu et al., 2007; Tian et al., 2019). Soybeans exhibit a unique plant architecture, as each node generates leaves, inflorescences, and pods; internode number greatly affects final soybean yields (Sun et al., 2019; Liu et al., 2020). It is currently difficult to achieve high yields by decreasing the number of nodes to reduce plant height and increasing the planting density in soybean. Several studies have shown that introducing the brachytic stem trait (shortened internodes with a zigzag arrangement of the main stem) into elite modern soybean varieties altered plant architecture to facilitate high‐density planting, reduce lodging, and increase yields (Adams and Weaver, 1998; Cui et al., 2007). Therefore, instead of changing the number of nodes, reducing plant height by shortening internodes to increase planting density may be an effective strategy for increasing soybean yields. To achieve this goal, we propose that soybean varieties with ideal plant architecture should have shorter internodes, more internodes, lodging tolerance, narrow leaflets, a higher proportion of four-seeded pods, smaller petiole angle, shorter petioles, and few or no short branches, allowing them to tolerate high‐density planting (**Figure 1**).

Modern crops have much lower genetic diversity than their wild relatives because artificial selection and population/genetic bottlenecks (Hyten et al., 2006; Lam et al., 2010; Qiu et al., 2017; Fernie and Yan, 2019). Wild species are rich sources of natural variation, which is important for improving the yield and quality of crops (Tian et al., 2019; Liu et al., 2021; Goettel et al., 2022; Huang et al., 2022). To understand the genetic architecture and networks underlying agronomic traits, it is crucial to isolate and characterize the genes responsible for plant architecture in soybean has been an important research topic for decades, but only a few genes controlling this trait have been characterized. Wild soybean represents an excellent germplasm resource for identifying key genes or alleles that could be used to develop high-yielding soybean varieties that tolerate dense planting via molecular breeding and gene editing.
